# Product Carbon Footprints and Their Uncertainties in Comparative Decision Contexts

**DOI:** 10.1371/journal.pone.0121221

**Published:** 2015-03-17

**Authors:** Patrik J. G. Henriksson, Reinout Heijungs, Hai M. Dao, Lam T. Phan, Geert R. de Snoo, Jeroen B. Guinée

**Affiliations:** 1 Institute of Environmental Sciences (CML), Leiden University, Leiden, the Netherlands; 2 Department of Econometrics and Operations Research, Faculty of Economics and Business Administration, VU University, Amsterdam, the Netherlands; 3 Department of Coastal Aquaculture, College of Aquaculture and Fisheries, Can Tho University, Vietnam; 4 Research Institute for Aquaculture No. 2, Department of Inland Resources & Fisheries Capture, Ho Chi Minh City, Vietnam; US Army Engineer Research and Development Center, UNITED STATES

## Abstract

In response to growing awareness of climate change, requests to establish product carbon footprints have been increasing. Product carbon footprints are life cycle assessments restricted to just one impact category, global warming. Product carbon footprint studies generate life cycle inventory results, listing the environmental emissions of greenhouse gases from a product’s lifecycle, and characterize these by their global warming potentials, producing product carbon footprints that are commonly communicated as point values. In the present research we show that the uncertainties surrounding these point values necessitate more sophisticated ways of communicating product carbon footprints, using different sizes of catfish (*Pangasius* spp.) farms in Vietnam as a case study. As most product carbon footprint studies only have a comparative meaning, we used dependent sampling to produce relative results in order to increase the power for identifying environmentally superior products. We therefore argue that product carbon footprints, supported by quantitative uncertainty estimates, should be used to test hypotheses, rather than to provide point value estimates or plain confidence intervals of products’ environmental performance.

## Introduction

Early enthusiasm about carbon footprinting resulted in the aim of calculating product carbon footprints (PCFs) for whole product assortments [1]. The conclusions were intended for industry to improve the product’s or service’s lifecycle environmental performance, and for consumers to encourage more sustainable product procurements. These ambitions soon floundered after being faced with the challenges of high costs of collecting data and modeling PCFs, large time investments, and a lack of consensus on modeling choices [1]. The 14067, 14040 and 14044 ISO standards for PCF and life cycle assessment (LCA), from which PCFs originate, provide the principles, minimum requirements and framework for conducting and reporting such studies [2–4]. ISO 14040, for example, defines the phases of LCAs: goal and scope definition, life cycle inventory analysis (LCI), life cycle impact assessment (LCIA) and interpretation [4]. In addition to ISO, numerous standards have been produced to harmonize methods based on the ISO standards [5,6]. Inventory databases and software solutions have also made it easier to calculate life cycle inventory results (e.g. kg CO_2_, CH_4_ and N_2_O), and classify and characterize these into PCFs (kg CO_2_-eq.). Results are commonly presented as absolute point values, which theoretically could be compared with each other much like nutritional facts [7]. Simply communicating the quantitative information through carbon labels has, however, been called into question, as consumers lack a daily or annual allowance for greenhouse gases (GHGs), unlike for nutrients [8].

Another reason for not communicating GHGs as point values is the large uncertainties surrounding these quantitative estimates. PCFs of identical products can deviate by an order of magnitude between studies, even if they comply with the same methodological guidelines [9]. This is largely due to data sourcing and modeling assumptions [9,10], but in some cases also to different characterization factors used to translate the environmental emissions into impacts [11]. The characterization factors for carbon footprints are typically the global warming potentials (GWPs 100-year) reported by the IPCC, based upon the radiative forcing of different gases.

LCA studies are often used for comparative purposes, including consumer choice. In a comparative context, two issues should be solved. The first is the fact that a standard LCA yields results on several impact categories, and that the trade-off between these categories is a delicate issue, requiring weighting and/or multi-criteria analysis [12,13]. The second is the fact that uncertainties in a comparative analysis require a different strategy, due to the fact that part of the uncertainty may be shared between the product alternatives [9]. In our work, we focus on the carbon footprint, so on just one category. Therefore the first issue is outside our scope. The second issue, however, is of central concern to us. While previous approaches dealt with shared uncertainties, they did not make the step to hypothesis testing, and neither to the implications for the labeling of individual products.

Despite the known limitations and uncertainties of PCF estimates, GHG savings have still made their way into regulations where they are enforced on a point-value basis. California’s Low Carbon Fuel Standard [14], for example, enforces 10% GHG savings for new fuels compared to a fossil fuel reference, and the EU’s fuel quality directive [15] uses a 6% margin.

Already in the 1990s were dispersion estimates made for a number of LCI related emission parameters [16,17]. Around the same time, there were also several new methodologies suggested for how to include quantitative uncertainties in life cycle inventories (LCIs)[18–21]. To date, however, the uncertainties considered have largely been limited to sensitivity analyses [22], default inventory ranges [23,24], characterization factors for one specific impact category [25,26], or pedigree estimates [27,28]. Pedigree estimates refer to a matrix of data quality indicators which evaluate the representativeness of the data used, which later are tentatively quantified using uncertainty factors based upon expert judgment or empirical data [27,29,30]. Statistical testing of outcomes, in the meantime, is rare among LCA studies, and where consulted it is largely limited to quotients (A/B) [31]. Table 1 summarizes a selection of LCA studies that take uncertainty into account. The table results show that this is the first study that evaluates empirical LCI uncertainty data, empirical LCIA uncertainty data, in a comparative analysis applying Monte Carlo dependent sampling and a hypothesis based significance test.

**Table 1:** A selection of LCA studies that take uncertainty into account, specifying if distributions are based upon real data (empirical) or upon default/pedigree estimates (conjectural), the propagation/sampling method used, if it is a comparative study, and in that case, if there is a hypothesis and any significance test carried out to test this hypnosis.

Reference	Input uncertainty data					Output results	
	Unit process data	Characterization factors	Propagation method	Comparative analysis	Sampling method	Hypothesis	Significance test
Basset-Mens et al 2009 [32]	Conjectural	No	Latin Hypercube	No	N/A	N/A	N/A
Bojacá and Schrevens 2010 [33]	Empirically based	No	Monte Carlo	No	N/A	N/A	N/A
Chen and Corson 2014 [34]	Partially Empirically based	N/A	Monte Carlo	Yes	Independent	None	N/A
Hauck et al 2014 [35]	Empirically based	Empirically based	Monte Carlo	Yes	Unknown	None	N/A
Heijungs and Kleijn 2001 [36]	Conjectural	Conjectural	Monte Carlo	Yes	Dependent	n(A>B) = n(A<B)	Runs test
Heijungs et al 2005 [37]	Conjectural	N/A	Taylor series	No	N/A	N/A	N/A
Heijungs et al 2005 [37]	Conjectural	N/A	Monte Carlo	Yes	Dependent	n(A>B) = n(A<B)	Runs test
Heijungs and Lenzen 2013 [38]	Conjectural	Conjectural	Taylor series	No	N/A	N/A	N/A
Heijungs and Lenzen 2013 [38]	Conjectural	Conjectural	Monte Carlo	Yes	Independent	None	N/A
Hong et al 2010 [39]	Conjectural	Empirically based	Taylor series	Yes	Independent	None	N/A
Hong et al 2010 [39]	Conjectural	Empirically based	Monte Carlo	Yes	Dependent	A/B = 1	N/A
Huijbregts et al 2003 [24]	Empirically based	Empirically based	Monte Carlo	Yes	Dependent	A/B = 1	N/A
Kennedy et al 1996 [28]	Conjectural	N/A	Monte Carlo	Yes	Independent	Median(A) = Median(B)	Tukey’s test
de Koning et al 2009) [9]	Conjectural	Conjectural	Latin hypercube	Yes	Independent	None	N/A
Lo et al 2005 [40]	Empirically based	Empirically based	Monte Carlo	Yes	Independent	None	N/A
Malça and Freire 2010 [41]	Meta-analysis	N/A	Monte Carlo	No	N/A	N/A	N/A
Mattila et al 2011 [31]	Empirically based	Yes, but source unknown	Monte Carlo	Yes	Dependent	A/B = 1	N/A
Maurice et al 2000 [42]	Largely Conjectural	No	Monte Carlo	Yes	Independent	None	N/A
Mutel et al 2013) [43]	Conjectural	Empirically based	Monte Carlo	Yes	Independent	None	N/A
Röös et al 2010) [23]	Conjectural	No	Monte Carlo	No	N/A	N/A	N/A
Röös et al 2011 [44]	Conjectural	No	Monte Carlo	No	N/A	N/A	N/A
Sonnemann et al 2002 [45]	Conjectural	No	Monte Carlo	Yes	Dependent	None	N/A
Steinmann et al 2014 [46]	Empirically based	Empirically based	Monte Carlo	No	N/A	N/A	N/A
Weber 2012 [47]	Meta-analysis	N/A	Monte Carlo	No	N/A	N/A	N/A
This study	Empirically based	Empirically based	Monte Carlo	Yes	Dependent	Median(A) = Median(B)	Wilcoxon

It is our belief that failure to explicitly and properly deal with uncertainties may result in counterproductive decisions, and that more extensive guidelines will merely reduce the number of flawed conclusions. Instead, the field of LCAs and PCFs needs to review some of the fundamentals of the scientific method, including statistically supported conclusions.

Statistically testing a hypothesis requires a predefined null hypothesis and quantification of uncertainties, two requirements that are rare in PCF studies. In comparative studies, the hypothesis conventionally presumes one product alternative to be better or equal to an alternative. The hypothesis is then critically evaluated using the appropriate statistical tests for the data under study. A product should consequently only be deemed beneficial if the null hypothesis can be statistically rejected.

Quantifying the dispersions around point values requires a variance and a distribution for unit process data and characterization factors, in addition to the central value (step 1). Next, a propagation method is needed [38]. In the present study Monte Carlo (MC) was used as it is the most commonly available propagation method and allows for post-hoc analyses. In a Monte Carlo, values are randomly sampled from the unit process distributions over a fixed number of iterations and aggregated into LCA results using an LCA matrix (step 2). This procedure produces a range of possible results, which in turn could be evaluated using different statistical tests and analyses (step 3). The outcomes are statistically supported environmental recommendations that can be communicated to policy makers or consumers through different channels (step 4).

**Fig 1:**
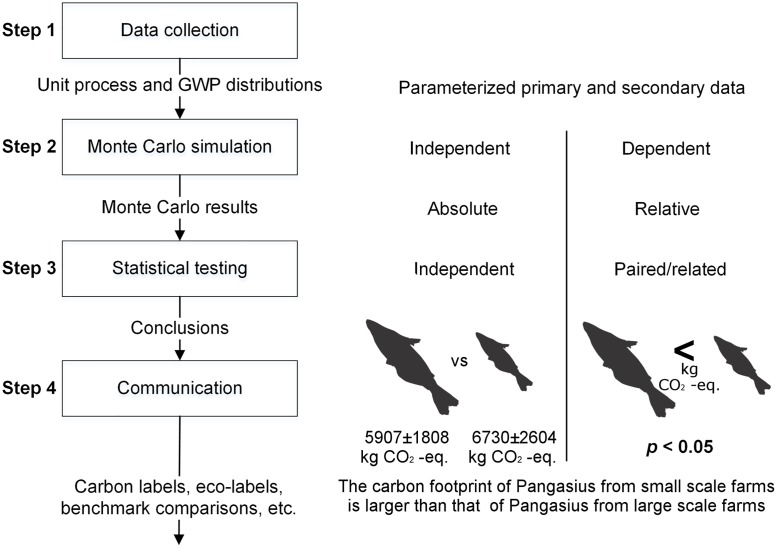
Procedures for propagating dispersions in data into product carbon footprints. PCFs can be propagated using either independent sampling yielding absolute results, or dependent sampling yielding relative results. For comparative purposes, dependent sampling is the only relevant option, and relative results can be a very useful way of presenting the LCA results for each sample. This also allows for paired statistical testing, increasing the probability of correctly rejecting the null hypothesis.

If results are to be used for comparisons, e.g. to decide if fish produced in larger corporate farms is better in terms of climate change impacts than fish produced in smaller family owned farms, the sampling procedure (step 2) for the products under study can be either dependent (correlated), where each product footprint builds upon the same sampled parameters, or independent (uncorrelated), where each product footprint builds upon a uniquely drawn set of random samples (Fig. 1) [36,39,48]. Independent sampling yields completely stochastic, incomparable results (“absolute results”), while dependent sampling produces results where all footprints are derived from the same set of sample values for both unit process data and characterization factors in each MC run. Thus, if the fish produced in larger corporate farms yield a very high outcome in a particular MC run, the fish produced in smaller family owned farms will most likely also yield a higher than average outcome, assuming that the two share many processes (e.g. electricity production, transportation processes, and disposal). Only the comparative difference between the results of each MC run, obtained by subtracting the sample result of one product from that of another, is therefore of importance in dependent sampling. We here label this as “relative results”. For comparative purposes, dependent sampling is the only relevant option, and relative results can be a very useful way of presenting the LCA results for each sample. In addition, relative results allow for powerful paired statistical testing of null hypotheses (step 3). The outcomes would, in turn, be communicated as one product being better than one or more alternatives (step 4).

In order to demonstrate the advantages of dependent sampling and to evaluate how to communicate PCFs with statistical tests, we use an LCA study of Vietnamese catfish (Pangasius spp.) fillets as an example [49]. The hypothesis explored was “Pangasius fish produced in larger corporate farms have smaller PCFs per unit of fish than those produced in smaller family-owned farms”. This hypothesis builds upon the assumption that corporations generally monitor and manage their farms better than family-owned farms and rely more heavily upon commercial feeds tailored to Pangasius fish. Thus, the null hypothesis tested assumed that the mean PCF of 36 randomly sampled family-owned farms would be equal to that of 36 corporate farms. While the absolute overall dispersions remain large, we managed to identify significant trend differences between the different farming systems by using our proposed approach.

## Methods

Data on the two farming systems and other related processes were collected between 2010 and 2013 as part of the EU FP7 SEAT project (S1–S3 Tables). Additional data were retrieved from the literature and the ecoinvent v2.2 database (www.ecoinvent.org). A complete description of the data used in the present research is available as supporting information (S1 Dataset) and in SEAT deliverable D3.5 [50]. Unit process distributions and variances were developed using the protocol presented in Henriksson et al. [30], reflecting inherent uncertainties (inaccuracies in measurements and models), spread (variability resulting from averaging) and unrepresentativeness (mismatch between the representativeness and use of data). The Anderson-Darling goodness-of-fit test was used to identify the distributions best representing data, limited to the four available distributions and generically assumed lognormal data in ecoinvent v2.2 [30].

The inventory flows were characterized using the GWPs and uncertainty distributions (S4 Table) reported in the fifth IPCC assessment report [51,52](step 1). In introducing uncertainties to GWPs, problems arise by the fact that the GWP of CO_2_ is 1 by definition (and thus has no uncertainty), while the GWPs of all other GHGs are normalized by that of CO_2_. Underlying GWPs (in kg CO_2_-eq. kg^-1^) are the absolute GWPs (AGWPs), which express the time-integrated radiative forcing (in W m^-2^ yr^-1^ kg^-1^) [51]. These AGWPs are uncertain, also for CO_2_. By adopting the uncertainty distributions on the level of GWPs we assume that these GWP uncertainties are based on dependent sampling of AGWPs in the models used by IPCC, e.g. dividing the AGWP for CH_4_ in each run by the AGWP for CO_2_ in the same run, thus forming a distribution of GWPs for CH_4_ and a point value of the GWP for CO_2_. The fifth IPCC assessment report [52] does, to our knowledge, not specify if the uncertainty estimates in the GWP of GHGs have been obtained through dependent or independent sampling, but judging the values of the uncertainties, we believe that dependent sampling has been used, as it should have been. Based on this assumption and in order to stay close to the traditional carbon footprint, we choose to use the GWPs with related uncertainty information for our characterization calculations from the fifth IPCC report [51,52], thereby maintaining the relative units and hence calculating carbon footprints in kg CO_2_-eq. The standard deviations (σ) supporting these GWPs were back calculated from the 90% uncertainty ranges (σ = (P95-P05) / (2*1.645)) presented in the fifth IPCC report [51,52]. For more details, please see S4 Table and Myhre et al. [51].

Results were scaled to one tonne of fish and propagated over 1 000 MC simulations using dependent sampling (step 2) and the matrix-based algebra [53] implemented in the CMLCA v5.2 (www.cmlca.eu) software. Statistical tests were conducted in SPSS (v.21).

**Fig 2:**
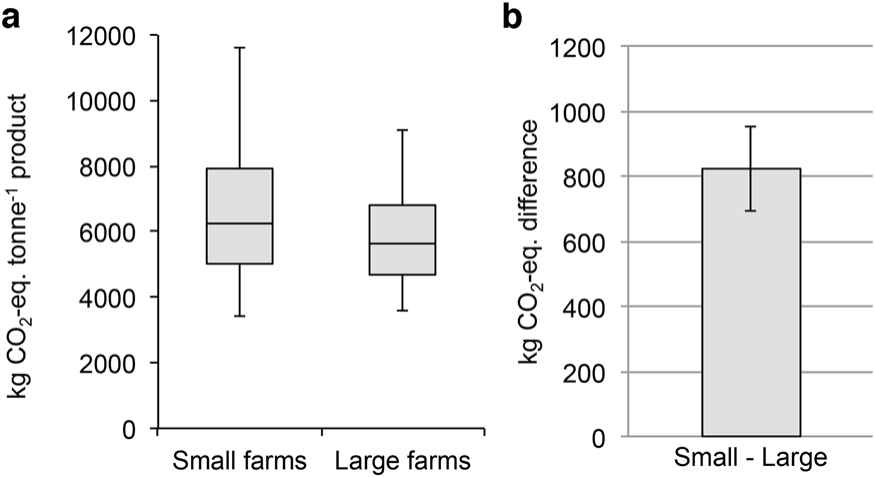
Greenhouse gas emissions resulting from the production of one tonne of Pangasius fish in small and large farms. (a) Box-and-whisker plot displaying the GHG emissions associated with fish from small (n = 36) and large (n = 36) sized Pangasius farms. Indicated are the median, the 25th percentile and 75th percentile (box), and the 10th and 90th percentiles (whiskers). (b) Median difference between fish from small and large farms on a per MC run basis, subtracting the GHG from the large farms from that of the small farms. Error bars indicate the 95% confidence interval of the median differences.

Of the two groups, family-owned farms were more reliant on farm-made feeds and agricultural byproducts (31% of all feeds) than large corporate farms, which almost exclusively (94%) relied upon commercial feeds (Fig. 2). Apart from feeds, all other supporting processes differed only in quantity, meaning that they rely upon the same shared supply chain, and hence on the same drawn values in each MC run, as well as stochastic GWPs. Emissions resulting directly from the fish ponds, however, were not shared between the two farming practices and therefore resulted in independently sampled values. For a more complete list of the data used and more specific results, see the supporting information to this article.

## Results

Both ranges of results were associated with large dispersions (S1 Fig.). From these, the mean difference between the two farming practices could be found by subtracting the result for fish from large corporate farms from that of fish from small family-owned farms for each MC run (Fig. 2). The mean difference between results did not follow a normal distribution and we therefore tested the median difference using the non-parametric one-sample Wilcoxon Signed Rank test (step 3), showing a highly significant (p < 0.001) difference of 824 kg CO_2_-eq. (see Fig. 2), thus indicating a significantly larger median PCF for fish from family-owned farms compared to fish from corporate farms (step 4).

## Discussion

As inventory models are data limited, most data supporting PCFs are opportunistically collected, rather than following a random sampling design. Concepts such as experimental design and statistical inference are therefore largely ignored in most footprinting exercises. Modeling choices also influence outcomes, including the choices of emission models, model structure, and mathematical equations. Product footprints are consequently influenced by conscious and unconscious choices, biasing statistical inference. Dependent sampling, however, reduces the effect of such choices, as the underlying choices remain largely consistent. The greater statistical power offered by paired statistical tests also reduces the risk of Type II statistical errors.

Only considering relative uncertainties is also favorable in situations where the origins of raw materials or products are untraceable. For example, aluminum derives from an energy intensive process and enters the global market from a pool of countries. The metal is then often traded, alloyed, worked up and assembled on geographically dispersed locations. The origin or origins of the aluminum raw material are therefore next to impossible to trace, while the resulting GHG emissions may differ with two orders of magnitude amongst different origins (e.g. China or Iceland) [52]. However, if only relative uncertainties are considered, the production of aluminum could be horizontally averaged to a global level while different aluminum products still could be compared with relatively high accuracy without simplifying the data.

Where requirements such as normally distributed populations and equal variances are fulfilled, a paired t-test is an appropriate test for comparing two products. However, in the case of a comparison involving three or more alternatives (e.g. small, medium, and large sized ponds), the paired comparison will not work due to the increased risk of type I errors. In such cases a test for related multiple comparisons should be used, two-way ANOVA being the most obvious choice, with an added Tukey test for post-hoc grouping into clusters of alternatives that differ significantly from one another. A non-parametric alternative for comparisons of more than two products is provided by the Friedman test. The clusters identified by the post-hoc test could serve as the basis for eco-labeling schemes, where each cluster represents a rank or a label (red, yellow or green), which easily could be communicated to e.g. consumers. Alternatively, a baseline product could be used for each product group (e.g. farmed salmon in the current example) to communicate results in ways more accessible to consumers.

## Conclusions

Product footprints were created to meet the need to steer our consumer society towards more sustainable choices. However, carbon footprints constitute a highly politicized field of science, where the decision stakes are high and system uncertainties large [53]. PCFs will therefore always be subject to intense scrutiny. In response, by re-evaluating PCFs as a strictly relative indicator while acknowledging the level of underlying uncertainty, clusters of environmentally superior products or production systems may be identified with a level of confidence. Our conclusions can be extended to other approaches for assessing products in a comparative sense, including the water footprint [54] and life cycle costing [55].

## Supporting Information

S1 Fig Histogram displaying the GHG emissions from the production of one tonne of Pangasius catfish in small and large scale ponds. (DOCX)

S1 Table Unit process data used for the Pangasius farms. The arithmetic mean was used as it is the expected central value of the CMLCA software.
(DOCX)

S2 Table Feed formula used for producing one tonne of commercial Pangasius feed in Vietnam (n = 4). From: Henriksson et al. (2014) Final LCA case study report—Primary data and literature sources adopted in the SEAT LCA studies. SEAT Deliverable D3.5—Annex report. Leiden, Netherlands.
(DOCX)

S3 Table Feed formula used for producing one tonne of farm-made Pangasius feed in Vietnam. From: Phan et al. (2009) Current status of farming practices of striped catfish, Pangasianodon hypophthalmus in the Mekong Delta, Vietnam. Aquaculture 296: 227–236.
(DOCX)

S4 Table Global warming potentials (GWPs) advocated in the fifth IPCC assessment report (2013). (DOCX)

S1 Dataset Unit process data, characterization factors and results of the present study. (XLSX)
