# Supplementary material for: Product Carbon Footprints and Their Uncertainties in Comparative Decision Contexts
Source: PLoS One. 2015 Mar 17;10(3):e0121221. doi: 10.1371/journal.pone.0121221 (PMC4363321; doi:10.1371/journal.pone.0121221)
Supplement: S1 Fig — (DOCX) [file pone.0121221.s001.docx]

S1 Fig. Histogram displaying the GHG emissions from the production of one tonne of Pangasius catfish in small scale and large scale ponds.
