# Supplementary material for: Product Carbon Footprints and Their Uncertainties in Comparative Decision Contexts
Source: PLoS One. 2015 Mar 17;10(3):e0121221. doi: 10.1371/journal.pone.0121221 (PMC4363321; doi:10.1371/journal.pone.0121221)
Supplement: S1 Table — The arithmetic mean was used as it is the expected central value of the CMLCA software. (DOCX) [file pone.0121221.s002.docx]

**Table S1: Unit process data used for the Pangasius farms.**The arithmetic mean was used as it is the expected central value of the CMLCA software.

|  |  | Small (n=36) | | | Large (n=36) | | |  |
| --- | --- | --- | --- | --- | --- | --- | --- | --- |
|  | Unit | Mean | C.V. | Distribution | Mean | Stdev | Distribution | Data source |
| Water area | m^2^ | 49.5 | 0.505 | Lognormal | 63.7 | 85.4 | Lognormal | [48] |
| Fingerlings | pcs | 1780 | 0.512 | Lognormal | 1980 | 1877 | Lognormal | [48] |
| Commercial feeds | t | 1325 | 0.294 | Normal | 1626 | 281 | Lognormal | [48] |
| Farm-made feeds | t | 502 | 0.500 | Lognormal | 111 | 240 | Lognormal | [55] |
| Agricultural by-products | t | 91 | 0.879 | Lognormal | 0 | 0 | n/a | [48] |
| Electricity | kWh | 44 | 1.432 | Lognormal | 54 | 83 | Lognormal | [48] & ecoinvent |
| Diesel | kg | 11.1 | 1.198 | Lognormal | 1.0 | 1.4 | Lognormal | [48] & ecoinvent |
