# Supplementary material for: Product Carbon Footprints and Their Uncertainties in Comparative Decision Contexts
Source: PLoS One. 2015 Mar 17;10(3):e0121221. doi: 10.1371/journal.pone.0121221 (PMC4363321; doi:10.1371/journal.pone.0121221)
Supplement: S2 Table — From: Henriksson et al. (2014) Final LCA case study report—Primary data and literature sources adopted in the SEAT LCA studies. SEAT Deliverable D3.5—Annex report. Leiden, Netherlands. (DOCX) [file pone.0121221.s003.docx]

**Table S2: Feed formula used for producing one tonne of commercial Pangasius feed in Vietnam (n=4).**
From: Henriksson et al. (2014) Final LCA case study report - Primary data and literature sources adopted in the SEAT LCA studies. SEAT Deliverable D3.5 – Annex report. Leiden, Netherlands.

| Name | Origin | Unit | Mean | CV | Distribution | Data used |
| --- | --- | --- | --- | --- | --- | --- |
| Fishmeal, domestic | VN | kg | 48 | 0.107 | Lognormal | [48] |
| Fishmeal, imported | PE | kg | 25 | 0.107 | Lognormal | [48] |
| Soybean meal | US | kg | 379 | 0.107 | Lognormal | [48] |
| Meat and bone meal | EU | kg | 34 | 0.107 | Lognormal | [48] |
| Blood meal | EU | kg | 1.0 | 0.107 | Lognormal | [48] |
| Rice bran | VN | kg | 253 | 0.099 | Lognormal | [48] |
| Cassava meal | VN | kg | 135 | 0.099 | Lognormal | [48] |
| Broken rice | VN | kg | 67 | 0.099 | Lognormal | [48] |
| Wheat flour | AU | kg | 58 | 0.099 | Lognormal | [48] |
| Fish oil, imported | PE | kg | 2,2 | 1.307 | Lognormal | [48] |
