# Supplementary material for: Product Carbon Footprints and Their Uncertainties in Comparative Decision Contexts
Source: PLoS One. 2015 Mar 17;10(3):e0121221. doi: 10.1371/journal.pone.0121221 (PMC4363321; doi:10.1371/journal.pone.0121221)
Supplement: S3 Table — From: Phan et al. (2009) Current status of farming practices of striped catfish, Pangasianodon hypophthalmus in the Mekong Delta, Vietnam. Aquaculture 296: 227–236. (DOCX) [file pone.0121221.s004.docx]

**Table S3: Feed formula used for producing one tonne of farm-made Pangasius feed in Vietnam.**
From: Phan et al. (2009) Current status of farming practices of striped catfish, Pangasianodon hypophthalmus in the Mekong Delta, Vietnam. Aquaculture 296: 227–236.

| Name | Origin | Unit | Mean | CV | Distribution | Data used |
| --- | --- | --- | --- | --- | --- | --- |
| Fishmeal | VN | kg | 93 | 0.629 | Lognormal | [48] |
| Low-value fish | VN | kg | 148 | 0.395 | Lognormal | [48] |
| Soybean meal | US | kg | 116 | 0.511 | Lognormal | [48] |
| Cassava meal | VN | kg | 115 | 0.565 | Lognormal | [48] |
| Rice bran | VN | kg | 394 | 0.394 | Lognormal | [48] |
| Cassava meal | VN | kg | 115 | 0.565 | Lognormal | [48] |
| Broken rice | VN | kg | 134 | 0.581 | Lognormal | [48] |
