# Supplementary material for: Product Carbon Footprints and Their Uncertainties in Comparative Decision Contexts
Source: PLoS One. 2015 Mar 17;10(3):e0121221. doi: 10.1371/journal.pone.0121221 (PMC4363321; doi:10.1371/journal.pone.0121221)
Supplement: S4 Table — (DOCX) [file pone.0121221.s005.docx]

**Table S4: Global warming potentials (GWPs) advocated in the fifth IPCC assessment report (2013)**

|  | GWP100 | | |
| --- | --- | --- | --- |
|  | Mean | SD | Distribution |
| CO_2_ | 1 | (0.16) | Normal |
| CH_4_ bio | 28 | 6.64 | Normal |
| CH_4_ fossil | 30 | 7.11 | Normal |
| N_2_O | 265 | 46.72 | Normal |
| NMVOC | 4.5 | 1.94 | Normal |
